# Work disability before and after a major cardiovascular event: a ten-year study using nationwide medical and insurance registers

**DOI:** 10.1038/s41598-017-01216-2

**Published:** 2017-04-25

**Authors:** Marianna Virtanen, Jenni Ervasti, Ellenor Mittendorfer-Rutz, Tea Lallukka, Linnea Kjeldgård, Emilie Friberg, Mika Kivimäki, Erik Lundström, Kristina Alexanderson

**Affiliations:** 1 Finnish Institute of Occupational Health, P.O. Box 40, FI-00251 Helsinki, Finland; 2 Karolinska Institutet, Department of Clinical Neuroscience, Division of Insurance Medicine, SE-171 77 Stockholm, Sweden; 3 Department of Public Health, University of Helsinki, Clinicum, P.O. Box 63, FI-00014 Helsinki, Finland; 4 Department of Epidemiology and Public Health, University College London, 1–19 Torrington Place, London, WC1E 7HB UK; 5 Karolinska Institutet, Department of Clinical Neuroscience, Division of Neurology, SE-171 77 Stockholm, Sweden

## Abstract

We examined the trajectories of work disability before and after IHD and stroke events. New IHD (n = 13521) and stroke (n = 7162) cases in 2006–2008 were retrieved from nationwide Swedish hospital records and their annual work disability days five years before and after the date of diagnosis were retrieved from a nationwide disability register. There was no pre-event differences in disability days between the IHD and stroke cases and five years prior to the event, they were close to those observed in the general population. In the first post-event year, the adjusted mean days increased to 83.9 (95% CI 80.6–86.5) in IHD; to 179.5 (95% CI 172.4–186.8) in stroke, a six-fold increase in IHD and 14-fold in stroke. Work disability leveled off among the IHD cases but not among those who had stroke. The highest disability levels for the fifth post-event year after a stroke event was associated with pre-existing diabetes (146.9), mental disorder (141.2), non-employment (137.0), and immigrant status (117.9). In a working-age population, the increase in work disability after a cardiovascular event decreases close ﻿﻿to the pre-event level in IHD but remains particularly high after stroke; among patients with comorbid depression or diabetes, immigrants, and those not in employment.

## Introduction

Ischemic heart disease (IHD) and cerebrovascular diseases (e.g., stroke) are the leading causes of disability and premature death worldwide^1, 2^. Of the working-age population in the European Union, approximately 24% have a longstanding illness and about 900 000 people die each year at working age^3^. CVD ranks high as a cause of death and years lost due to disability^3^. However, during past decades, new invasive and other medical treatments have considerably improved the prognosis of CVD^4, 5^, increasing work participation among survivors.

Previous studies on work disability associated with CVD have usually focused on clinical samples and the post-event return to work. They have shown that a majority of the survived IHD patients and approximately half of the stroke patients return to work during the first year following the event^6–9^. A few studies have examined changes in work disability levels around the event^10–13^. In one study the data were limited to high-risk patients treated for hyperlipidemia^10^ and other studies have examined disability either before CVD death^11^ or after coronary revascularization procedures^12, 13^. Furthermore, although IHD and stroke might have different impact on disability due to their different prognosis^14^, previous studies have not compared the pre- and post-event work disability levels between these diseases. Also the contribution of important risk factors such as socioeconomic factors and co-occurring diseases is not known.

In this study, we assessed five-year pre- and post-event work disability levels comparing individuals with the onset of IHD to those with stroke. We also evaluated the risk factors that have rarely been examined but potentially contribute to work disability before and after the onset of disease, such as education, immigration status and comorbid diseases, using a nationwide register including all people of working–age living in Sweden.

## Results

### Descriptive characteristics

As shown in Table 1, proportionally more IHD cases than stroke cases were older, male, less educated, married or cohabiting, born outside Sweden, and lived in rural areas. People with stroke were more likely to be non-employed, previously have received specialized in- or outpatient care for a mental disorder and have cancer, while those with IHD were more likely to have diabetes. Of the IHD cases, 15.9% underwent coronary revascularization during the last pre-event or first post-event year. An intravenous intracranial procedure was applied to 3.3% of the stroke patients.

**Table 1:** Descriptive characteristics at time of event among a cohort with newly diagnosed ischemic heart disease (IHD) or stroke.

Characteristics at time of event	All (n = 20683)	IHD (n = 13521)	Stroke (n = 7162)	*P*-value for difference*
Age; mean (SD)	52.3 (6.9)	53.0 (6.2)	51.0 (8.0)	<0.0001
Sex: men	15347 (74.2)	10780 (79.7)	4567 (63.8)	<0.0001
women	5336 (25.8)	2741 (20.3)	2595 (36.2)	
Education: low	5040 (24.4)	3450 (25.5)	1590 (22.2)	<0.0001
intermediate	10351 (50.1)	6749 (49.9)	3602 (50.3)	
high	5292 (25.6)	3322 (24.6)	1970 (27.5)	
Family situation: married or cohabiting	12607 (61.0)	8467 (62.6)	4140 (57.8)	<0.0001
not married or cohabiting, no children in household	6773 (32.8)	4298 (31.8)	2475 (34.6)	
not married or cohabiting, children in household	1303 (6.3)	756 (5.6)	547 (7.6)	
Birth country: Sweden	17357 (83.9)	11194 (82.8)	6163 (86.1)	<0.0001
other	3326 (16.1)	2327 (17.2)	999 (13.9)	
Type of living area: large city	6840 (33.1)	4324 (32.0)	2516 (35.1)	<0.0001
medium-sized town	7279 (35.2)	4764 (35.2)	2515 (35.1)	
small town/village	6564 (31.7)	4433 (32.8)	2131 (29.8)	
Economic activity: employed	18191 (88.0)	11947 (88.4)	6244 (87.2)	0.013
other	2492 (12.1)	1574 (11.6)	918 (12.8)	
Mental disorder: no	19301 (93.3)	12698 (93.9)	6603 (92.2)	<0.0001
yes	1382 (6.7)	823 (6.1)	559 (7.8)	
Cancer: no	20145 (97.4)	13206 (97.7)	6939 (96.9)	0.0008
yes	538 (2.6)	315 (2.3)	223 (3.1)	
Diabetes: no	19172 (92.7)	12364 (91.4)	6808 (95.1)	<0.0001
yes	1511 (7.3)	1157 (8.6)	354 (4.9)	
Coronary revascularization/intravenous intracranial procedure: no	18294 (88.5)	11369 (84.1)	6925 (96.7)	<0.0001
yes	2389 (11.6)	2152 (15.9)	237 (3.3)	

Figures are number (%) unless otherwise stated.

*P-value for difference between IHD and stroke cases.

### Pre- and post-event trends in disability

**Figure 1:**
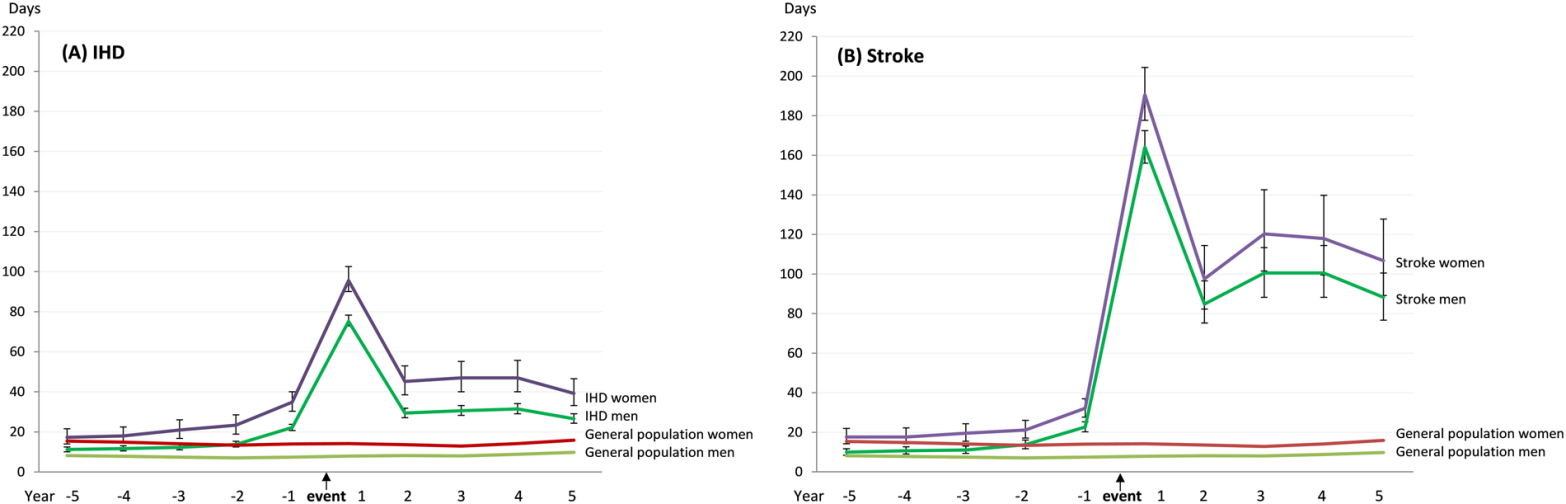
Mean days (95% confidence intervals) of work disability among men and women 5 years before and 5 years after newly diagnosed cardiovascular disease and men and women in the general population; IHD (Panel A) or stroke event (Panel B), unadjusted estimates*. *Adjusted estimates are shown in Supplementary Figure 2.

As shown in Fig. 1, women generally had higher levels of disability than men throughout the study period. In both IHD and stroke, the overall mean number of sickness absence days increased as the date of event approached: pre-event range in IHD was 11.2 to 22.2 among men; 17.3 to 34.8 among women; in stroke 10.0 to 22.6 among men; 17.6 to 32.1 among women. The largest increase during the pre-event period was seen in the last year prior to the event. The corresponding mean number of sickness absence and disability pension days among the general population was rather stable, varying between 7.1 and 9.8 (men) and between 12.9 and 15.9 (women) over the corresponding 10-year study period. Thus, the mean work disability days among IHD and stroke cases were close to the general population five years before the event, but began to increase thereafter. Age- and education-adjusted mean number of disability days among men and women are shown in Supplementary Figure 2, with no remarkable differences with the unadjusted figures shown in Fig. 1. The estimates with men and women combined are as follows: IHD: mean 14.2, 95% CI 12.7–15.8 in year -5, mean 27.4, 95% CI 25.5–29.4 in year -1; stroke: mean 12.7, 95% CI 11.1–14.6 in year -5, mean 27.1, 95% CI 24.8–29.7 in year -1; adjusted for age, sex and education). During the first post-event year, a large, rapid increase emerged in both disease groups, also showing a vast difference between the IHD and stroke cases (Fig. 1 and Supplementary Figure 2). The age, sex and education adjusted mean number of disability days during the first post-event year was 83.9 (95% CI 80.6–86.5) among the IHD cases, and 179.5 (95% CI 172.4–186.8) among the stroke cases. Thus, we observed an approximately six-fold increase in work disability associated with the onset of IHD, and a 14-fold increase associated with stroke. By the second post-event year, work disability days levelled off among the IHD cases, returning to levels close to those observed in the pre-event year (mean 31.2, 95% CI 28.5–34.1 in year 5 after the event). However, among the stroke cases, the mean number of work disability days remained high (mean 99.5, 95% CI 89.1–111.1). Multivariable adjusted estimates were similar (data not shown).

The annual relative difference in work disability days between IHD and stroke cases is shown in Supplementary Table 1. People who had a stroke event had 2.15- to 3.20-fold higher level of work disability days compared to people with IHD during all post-event years. This difference increased from 2.15 (2.04–2.26) in the first post-event year to 3.20 (2.80–3.66) in the fifth post-event year.

**Figure 2:**
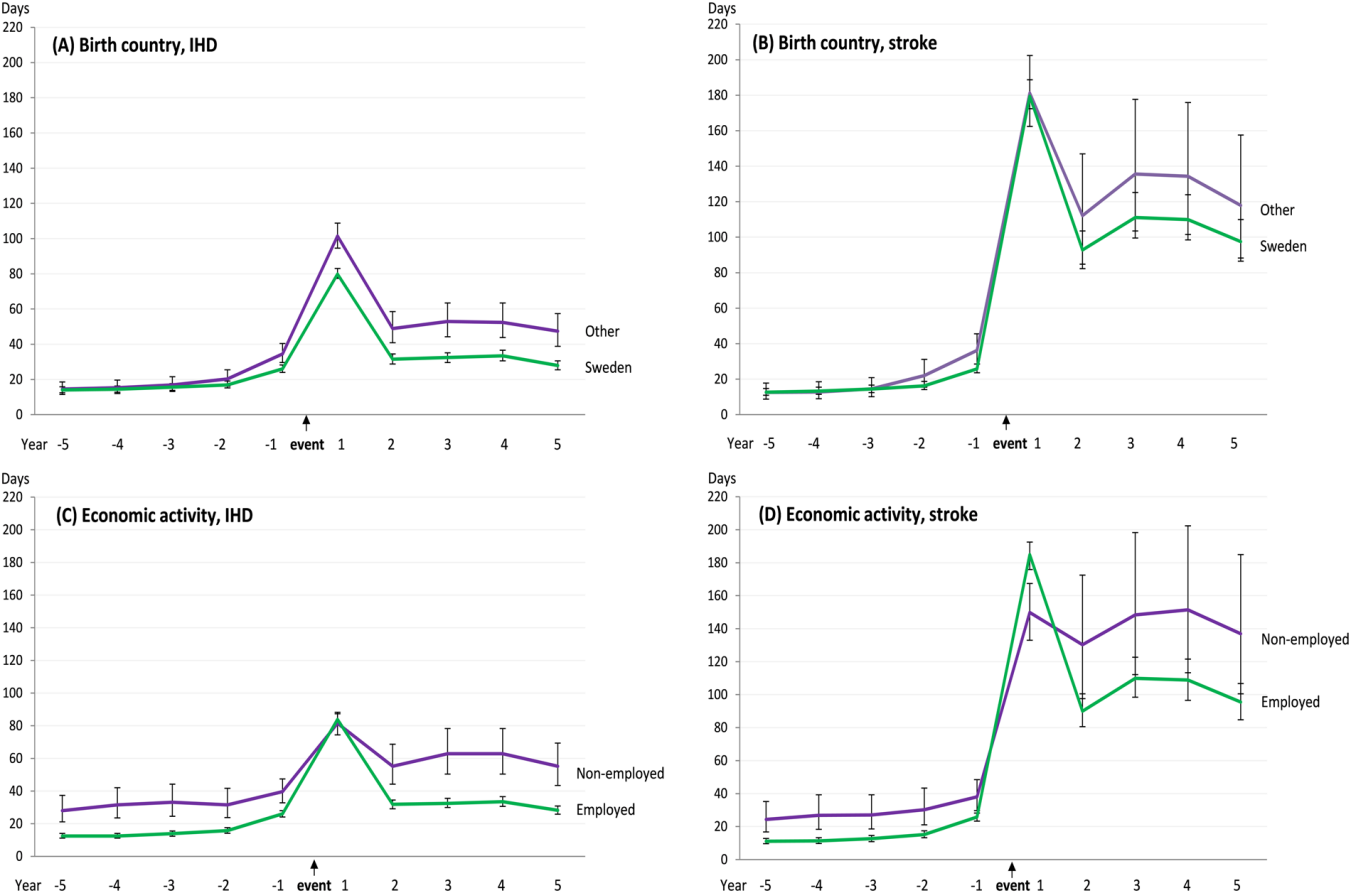
Mean days (95% confidence intervals) of work disability 5 years before and 5 years after newly diagnosed ischemic heart disease (IHD) or stroke event, by birth country and economic activity (adjusted for age, sex, and education).

We then examined pre- and post-event trends in work disability by socio-demographic characteristics and comorbid diseases. There was no difference between the pre-event sickness absence days five to two years prior to the event among those who were born in Sweden and those who were not (Fig. 2, Panels A and B), but in the year preceding the event, the sickness absence days increased more among those not born in Sweden (RR = 1.32 higher for non-Swedish-born among those who had IHD in the following year and RR = 1.40 among those who had stroke). During the first post-event year, the difference between foreign-born and Swedish born disappeared among the stroke cases, but thereafter, foreign-born had higher levels of disability associated with both IHD and stroke. The risk of sickness absence among the non-employed was approximately twice that of the employed during the pre-event period, a similar difference to that seen among the IHD and stroke groups (Fig. 2, Panels C and D). This difference disappeared during the first post-event year (among the stroke cases, the non-employed actually had a lower rate of work disability compared to the employed (RR = 0.81). As of the second post-event year, the difference between the non-employed and employed re-emerged among both IHD and stroke cases.

**Figure 3:**
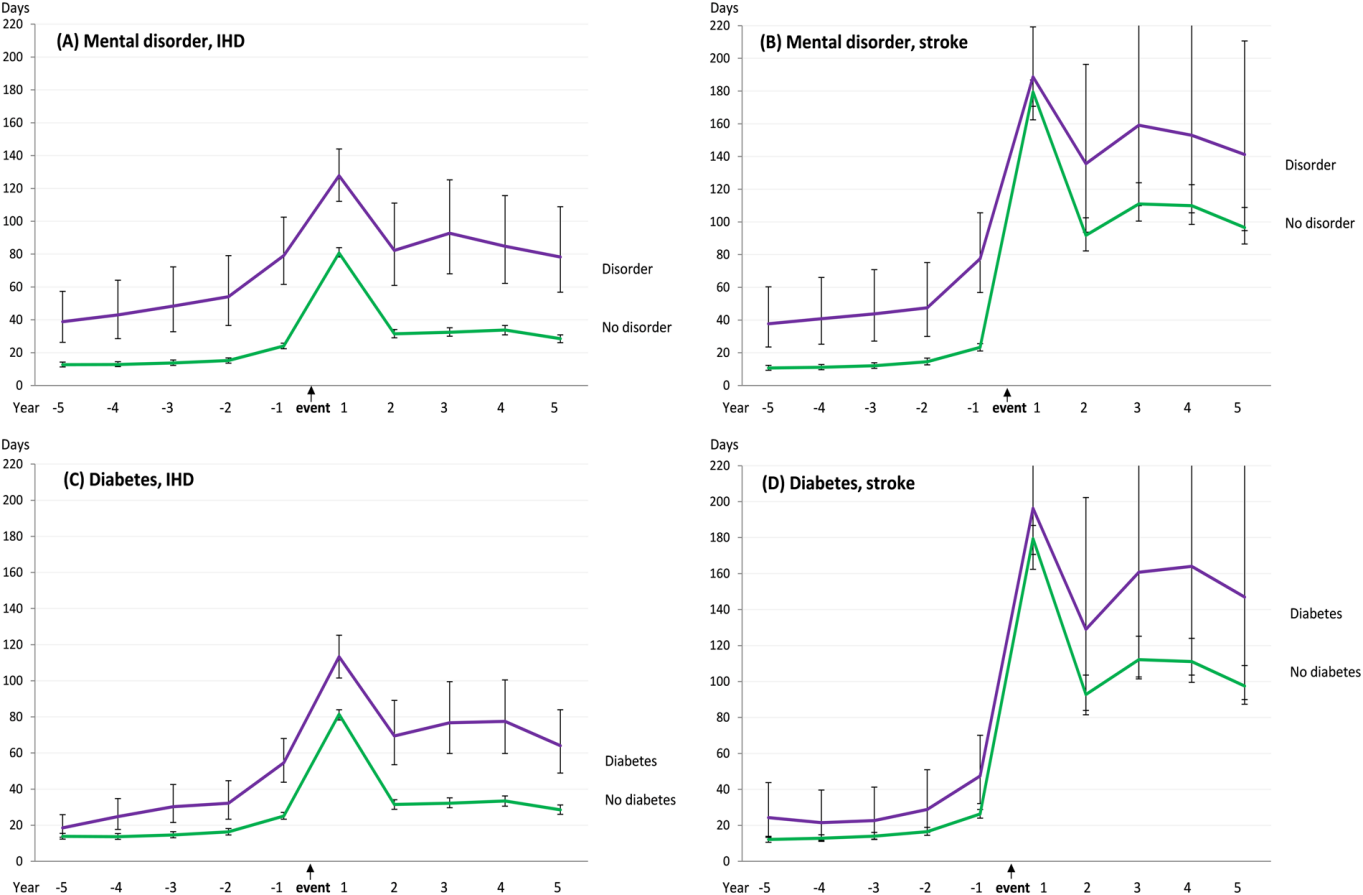
Mean days (95% confidence intervals) of work disability 5 years before and 5 years after newly diagnosed ischemic heart disease (IHD) or stroke event, by mental disorder and diabetes status (adjusted for age, sex, and education).

Having a mental disorder was associated with more than a 3-fold risk of sickness absence during the whole pre-event period among IHD and stroke cases (Fig. 3, Panels A and B). During the first post-event year, this difference decreased among the IHD cases (RR = 1.58) and disappeared among the stroke cases (RR = 1.05). From the second post-event year onwards, the difference returned close to the level seen during the last pre-event year among the IHD but not among stroke cases. A comparison between people with and without diabetes revealed a rather similar pattern than that found for mental disorders, except that the pre-event differences were smaller (Fig. 3, Panels C and D).

The trends for other socio-demographic factors (Supplementary Figures 3 and 4) suggest that the differences in family situations and type of living area were small, while for older individuals (>50 years), the mean post-event work disability days remained relatively high among stroke cases. Educational differences remained relatively stable during follow-up.

In absolute terms the highest disability levels for the fifth post-event year from a stroke was seen among people with a pre-existing diabetes (mean days 146.9, 95% CI 90.0–242.3) or mental disorder (141.2, 95% CI 94.6–210.6), among the non-employed (137.0, 95% CI 100.5–184.9) and immigrants (117.9, 95% CI 88.2–157.6). We found significant interactions between event type (IHD versus stroke) and the following baseline characteristics associated with the level of mean work disability days; birth country, economic activity, mental disorder and diabetes in the first post-event year (*P* = 0.002; *P* = 0.022; *P* < 0.0001; *P* = 0.036, respectively). For mental disorder, there was a significant interaction in all subsequent post-event years as well (*P* = 0.026, *P* = 0.007, *P* = 0.023, *P* = 0.020). The significant interactions support the finding which showed, for example, that the difference in work disability between patients with and without mental disorders almost disappeared among stroke patients during the first post-event year while among IHD patients, only a modest reduction was observed.

## Discussion

The present study examined ten-year trajectories of work disability around major cardiovascular events, IHD and stroke. With large complete nationwide data on newly diagnosed IHD and stroke cases, we included people who were of working age and not on disability pension. We were able to assess the course of work disability before and after the event, and to investigate the differences between sub-groups. The levels of sickness absence during the pre-event period were similar in IHD and stroke, and five years before the event, close to the level observed in the general population. The mean annual number of disability days increased to 27 days in the last year before the diagnosis date and peaked to 84 days (IHD) and 180 days (stroke) during the first post-event year. Thus, we observed a six-fold increase in work disability associated with the onset of IHD and a 14-fold increase associated with the onset of stroke. The excess risk of stroke compared to IHD only emerged after the event and remained two- to three-fold throughout the post-event period.

We found that the level of post-event work disability was higher among individuals with a stroke event compared to individuals with an IHD, which might be a reflection of the much poorer prognosis of stroke patients^14^. In this study, we examine IHD and stroke as prognostic factors in the labor market and work disability trends among people who were not permanently or long-term disabled at inclusion (i.e., not excluded from the labor market). Our findings confirm previous studies on clinical samples which have reported that more IHD patients than stroke patients return to work during the first post-event year^6^. With a long follow-up, we demonstrated that the substantial difference between the work disability levels of IHD and stroke patients persisted over the whole five-year post-event period. Among IHD cases, the level of work disability almost returned to the level seen in the pre-event year (a mean of 31 days a year), whereas among stroke cases, the disability rates remained high (100 days). The reasons for this discrepancy might be related to clinical factors such as the substantially poorer functional capacity and prognosis of stroke sufferers than those with IHD, as well as the psychosocial factors that differently affect people with IHD and stroke, such as depression, increased social isolation, increased barriers to return to work, and working conditions that are not adjustable to stroke survivors’ impaired work capacity^6, 9, 15^. Nevertheless, even in the first post-event year, the mean number of work disability days remained well below the possible 365 days, indicating that the whole post event year was not spent disabled to work.

In our sub-group analyses, the highest absolute post-event work disability levels were found among people with stroke who also had a pre-existing diabetes or mental disorder, and among those who were non-employed, for example, unemployed or otherwise not engaged in paid work at the time of the event. However, in stroke analyses, the confidence intervals were wide; thus the differences in absolute levels can only be regarded as explorative.

A general observation was a reduction or even a disappearance of sociodemographic and health-related differences during the first post-event year, in particular among the stroke cases. As the disease in its acute phase is generally highly disabling, the causes behind the inequalities may play a smaller role. The reasons why work disability levels vary between groups might have a different importance depending on the phase of the disease; for example, the delivery and quality of healthcare and rehabilitation provided, financial resources supporting recovery and rehabilitation, social support and networks, lifestyle, self-care capacities, and adherence^5^. As the present population comprised those who survived at least 30 days after the event, our results did not account for socially patterned differences in immediate or rapid post-event mortality^5^.

The difference in work disability between the foreign-born compared with the Swedish-born did not emerge until the last year before the event. A previous study has shown that a higher risk of work disability among immigrants is indeed attributable to their lower occupational grade and income, suggesting no overall association after adjustment for these^16^. In our study, the difference between foreign-born and Swedish-born was robust to adjustment for socioeconomic status. However, our findings are particularly relevant in that they show a more detailed pattern of the work disability associated with the onset of CVD, as we demonstrated that a CVD event generates differences in ability to work between those born in Sweden and elsewhere^5^.

People with pre-existing mental disorders had over three times more sickness absence days during the pre-event period, among both IHD and stroke cases, which was the largest relative risk observed in this study. Mental disorders and diabetes also constituted the highest absolute level of work disability during the later post-event years. These findings are in accordance with a high global burden of diabetes and mental disorders, as expressed by years lost due to disability or early death^2, 17^. Mental disorders, particularly depression, associated with an IHD or stroke event might worsen the prognosis by, for example, precluding the patient from participating in physical rehabilitation and cognitive therapies, adhering to medical treatments, or making the necessary lifestyle changes needed to achieve full functional recovery after the event^15, 18, 19^. Comorbid diabetes, in turn, might be associated with more severe disease, complications, and poorer prognosis^20, 21^.

The major strengths of register-based studies are their high coverage and specificity^22^ and no loss to follow-up, but they also have some limitations. Those data typically comprise a limited set of potential confounding factors; for example, information on health behaviors and severity of illness was not available. We adjusted the models for medical treatment procedures received, which can be considered a proxy for severity of illness, and we excluded angina cases, milder forms of IHD^23^. We did not have data on health behaviors which, however, have shown to contribute relatively little to work disability in cardiometabolic diseases, whereas the contribution of comorbid diseases, especially mental disorders, has been substantially high^24^. Another register-based limitation is that we did not have data on work disability episodes that lasted less than 15 days (i.e., we only had days covered by the Social Insurance Agency). However, as our data covered the whole country, these findings are directly generalizable to the Swedish working-age population, and possibly to other societies with similar social security and healthcare systems.

In conclusion, the onset of IHD was associated with a six-fold and stroke with a 14-fold increase in work disability after the event. Much of this increased work disability levelled off among the IHD patients, but not among the stroke patients, suggesting a more positive prognosis for ability to work for IHD. The highest post-event work disability levels were found among people who had a stroke and additionally had a pre-existing mental disorder or diabetes and among those who were non-employed or immigrants. Further investigations are required to identify measures to improve working capacity after major CVD events, particularly stroke.

## Data and Methods

Data were retrieved from nationwide register linkage of the Insurance Medicine All-Sweden (IMAS) study^25–27^. The study population was based on several national registers linked for research purposes. Ethical vetting is always required when using register data in Sweden and performed by regional review boards, and the risk appraisal associated with the Law on Public Disclosure and Secrecy is performed by data owners (register keepers). The ethical review boards can waive the requirement to consult the data subjects directly to obtain their informed consent, and often does so if the research is supported by the ethical review board and the data have already been collected in some other context (e.g., insurance records). Also, the institutional review board/ethics committee waived the need for written informed consent from the study subjects. All registers were anonymized and de-identified prior to analysis by the authority, Statistics Sweden, which was responsible for data linkage and researchers received de-identified data. According to these standards in Sweden, this project has been approved by the Regional Ethical Board of Karolinska Institutet, Stockholm, Sweden. The study population consisted of all individuals with a newly diagnosed CVD event (IHD or stroke) in 2006, 2007, or 2008, who were of working age (25 to 60 years) in the event year, had been living in Sweden since 31th December 2000, had no indication of cardiovascular events registered in Sweden between 2001 and the event year, and survived 30 days after the event (see Supplementary Figure 1). This resulted in a cohort of 28 374 individuals. Those with both IHD and stroke were excluded (n = 137) as well as those who were on permanent disability pension or had an ongoing sick-leave spell that lasted for more than 730 days (i.e., 2 years; n = 7554), thus being out of the labor market. The analytic sample consisted of 20683 individuals (13521 with IHD and 7162 with stroke). Work disability data for the pre-event period were available for all and for the post-event period, either until death or emigration which occurred for 1401 individuals, or until the end of follow-up (5^th^ post-event year). For comparison, we calculated the mean days of work disability for the total population with similar criteria (n = 3 668 634–3 601 882) over a 10-year period (2003–2012).

### Case definitions

The National Board of Health and Welfare’s hospitalization register provided data on incident non-fatal IHD events (International Classification of Diseases, ICD-10 codes I21–I25 for myocardial infarction or other IHD). Incident non-fatal stroke was based on hospitalization for stroke (ICD-10 codes I60, I61, I63, I64).

### Work disability

Work disability was defined as the annual number of compensated absence days from work due to sickness (before the event) or sickness and disability pension (after the event) for each individual. These data were obtained from the National Social Insurance Agency. In Sweden, all people with income from work or unemployment benefits who have reduced capacity for work due to a disease or injury are eligible for sickness benefits. For employed, the first 14 days of a sick-leave spell are paid by the employer, and thus not included in the Social Insurance Agency data. All, also those with no previous income, can be granted disability pension if the capacity for work is deemed to be permanently reduced.

### Covariates

The following covariates were assessed at the end of the year preceding the event and retrieved from Statistics Sweden’s Longitudinal Integration Database for Health Insurance and Labour Market Studies (LISA): age; sex; educational level (‘low’ (0–9 years), ‘intermediate’ (10–12 years), and ‘high’ (≥13 years); family situation (‘married or cohabiting’, ‘not married or cohabiting, without children’ (i.e., living alone), ‘not married or cohabiting but with children’ (i.e., single parent); country of birth (Sweden vs. other); type of living area (‘large city’, i.e., Stockholm, Gothenburg and Malmö, ‘medium-sized town’, ‘small town/village’); and economic activity (currently in employment, including self-employed vs. those not in employment, i.e., unemployed, on parental leave, student, etc.). Comorbid diseases included cancer (ICD-10 codes C00-D48), based on the Swedish cancer register; mental disorders (F00-F99), and diabetes (E10-E14), based on information from the specialized care in- and outpatient patient register covering the year before the event date. Medical treatment procedures for CVD (a year prior to the event to the year after the event) included coronary revascularization (coronary artery bypass graft, percutaneous transluminal coronary angioplasty, other coronary distension procedure), or intravenous intracranial procedure (operation for aneurysm, other intracranial vein operations, other endovascular procedures), and were also based on the in- and outpatient register. Treatment procedures were assessed to control variation in the severity of illness as well as their possible effect on prognosis.

### Statistical analysis

We compared the baseline descriptive characteristics of the IHD and stroke cases, using the χ^2^ test for categorical variables and ANOVA for age. We then examined the five-year work disability levels for IHD and stroke cases before and after the event by calculating the least square means of annual days of work disability. From least square means we further calculated their exponential functions and their 95% confidence intervals (CI). The differences between the IHD and stroke cases were expressed as rate ratios (RR) and their 95% confidence intervals (CI). The models were first adjusted for sociodemographic factors (age, sex, education) and then for other covariates (family situation, birth country, type of living area, economic activity, comorbid disease [mental disorder, diabetes, or cancer], and treatment procedure). Using a similar method, we calculated the mean annual days of work disability for the subgroups of covariates (e.g., sex, educational level, comorbid disease [except cancer in which the number of cases was low]). Interactions between IHD versus stroke, and the covariates were tested for each study year, to assess whether the type of disease (IHD, stroke) modified the association between the covariates and annual work disability days. This approach enabled us to identify potential risk groups for increased or prolonged work disability associated with an IHD or stroke. SAS version 9.4 (SAS, Cary, NC, USA) was used for all analyses.

## Electronic supplementary material

Online Supplement
