# Supplementary material for: Work disability before and after a major cardiovascular event: a ten-year study using nationwide medical and insurance registers
Source: Sci Rep. 2017 Apr 25;7:1142. doi: 10.1038/s41598-017-01216-2 (PMC5430721; doi:10.1038/s41598-017-01216-2)
Supplement: Supplementary file 1 — Online Supplement [file 41598_2017_1216_MOESM1_ESM.pdf]

ONLINE SUPPLEMENT

Work disability before and after a major cardiovascular event: a ten-year study using nationwide medical and insurance registers

Marianna Virtanen, Jenni Ervasti, Ellenor Mittendorfer-Rutz, Tea Lallukka, Linnea Kjeldgård, Emilie Friberg, Mika Kivimäki, Erik Lundström, Kristina Alexanderson

Supplementary Table 1. Comparison of work disability rates between ischemic heart disease (IHD) and stroke cases during 5-year pre-event and 5-year post-event period

	Year in relation to event<br>Rate ratio (95% confidence interval)*									
	**-5**	**-4**	**-3**	**-2**	**-1**	**+1**	**+2**	**+3**	**+4**	**+5**
IHD	1.00	1.00	1.00	1.00	1.00	1.00	1.00	1.00	1.00	1.00
Stroke	0.90 (0.76-1.06)	0.91 (0.77-1.08)	0.91 (0.77-1.08)	0.97 (0.83-1.14)	0.99 (0.89-1.11)	2.15 (2.04-2.26)	2.75 (2.42-3.12)	3.19 (2.82-3.62)	3.08 (2.71-3.51)	3.20 (2.80-3.66)

*Adjusted for age, sex, and education.

Supplementary Figure 1. Study design and selection of study sample

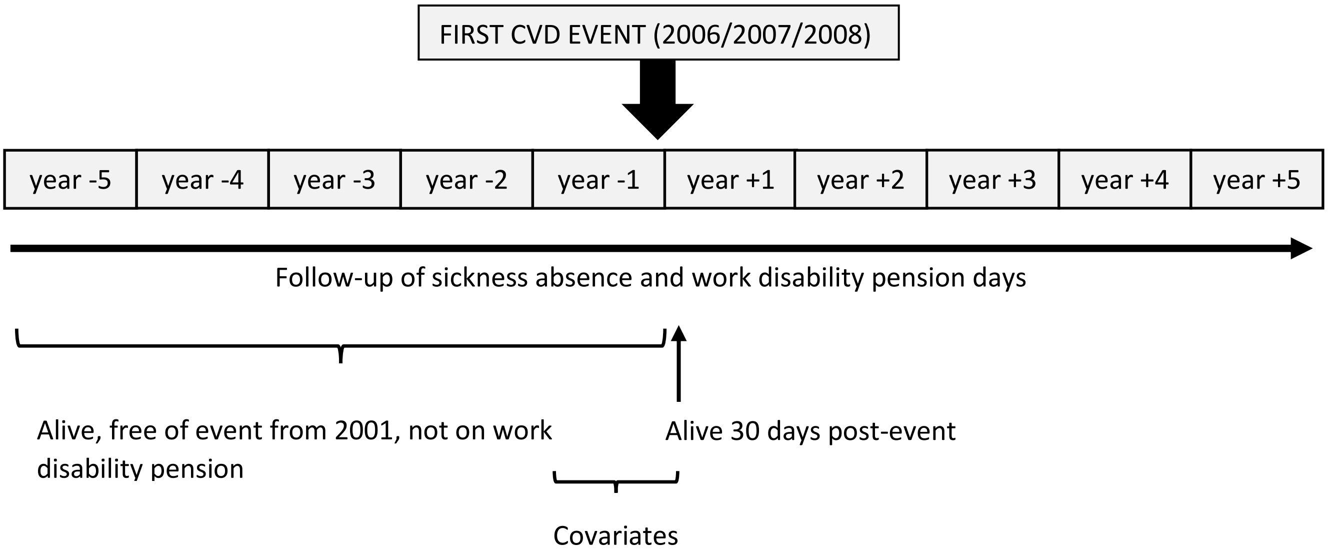

Supplementary Figure 2. Adjusted* mean days (95% confidence intervals) of work disability among men and women 5 years before and 5 years after newly diagnosed ischemic heart disease; IHD (Panel A) or stroke event (Panel B)

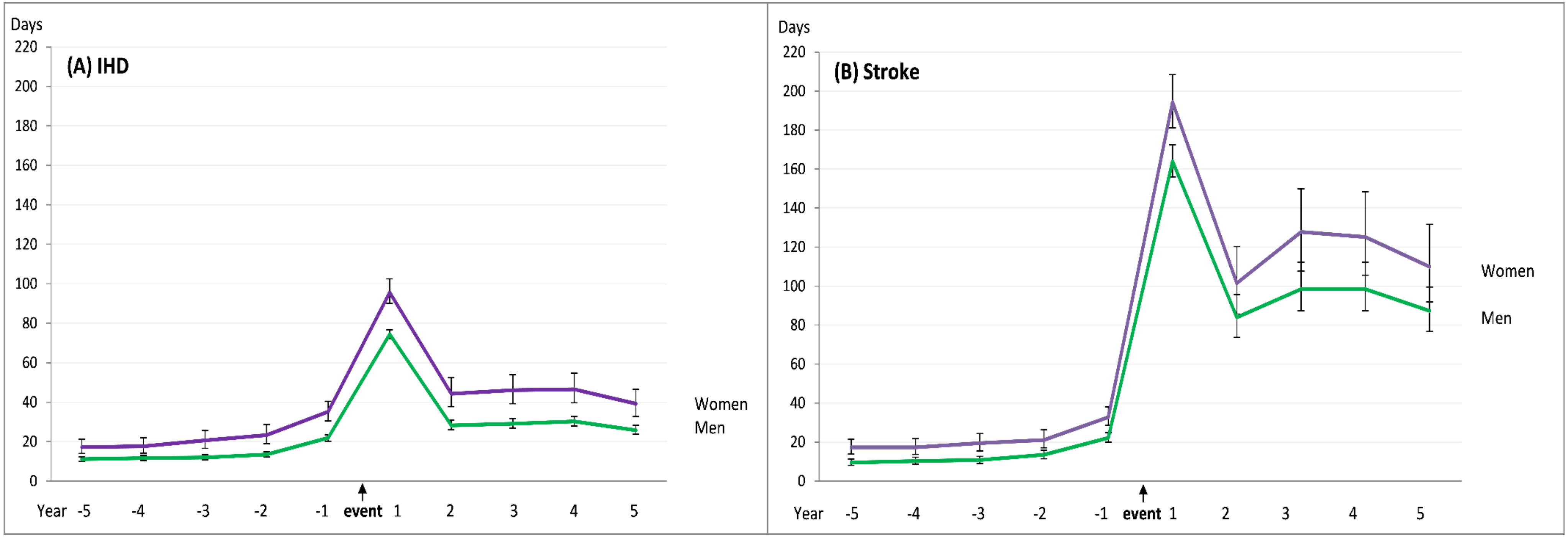

*Adjusted for age and education.

Supplementary Figure 3. Adjusted* mean days (95% confidence intervals) of work disability 5 years before and 5 years after newly diagnosed ischemic heart disease (IHD) or stroke event, by age and family situation

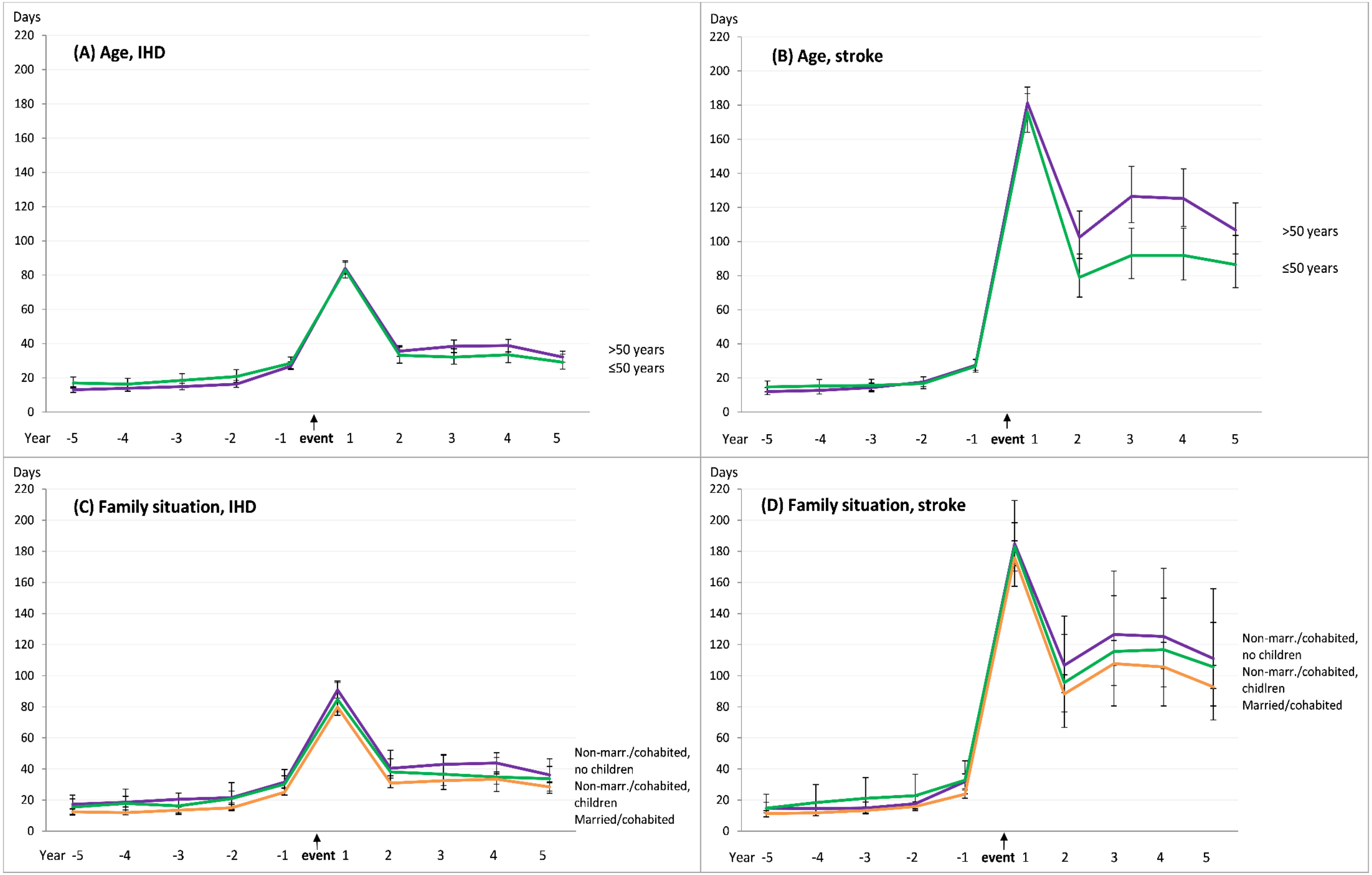

*Age-stratified analysis adjusted for sex and education; family situation stratified analysis adjusted for age, sex, and education.

Supplementary Figure 4. Adjusted* mean days (95% confidence intervals) of work disability 5 years before and 5 years after newly diagnosed ischemic heart disease (IHD) or stroke event, by type of living area and educational level

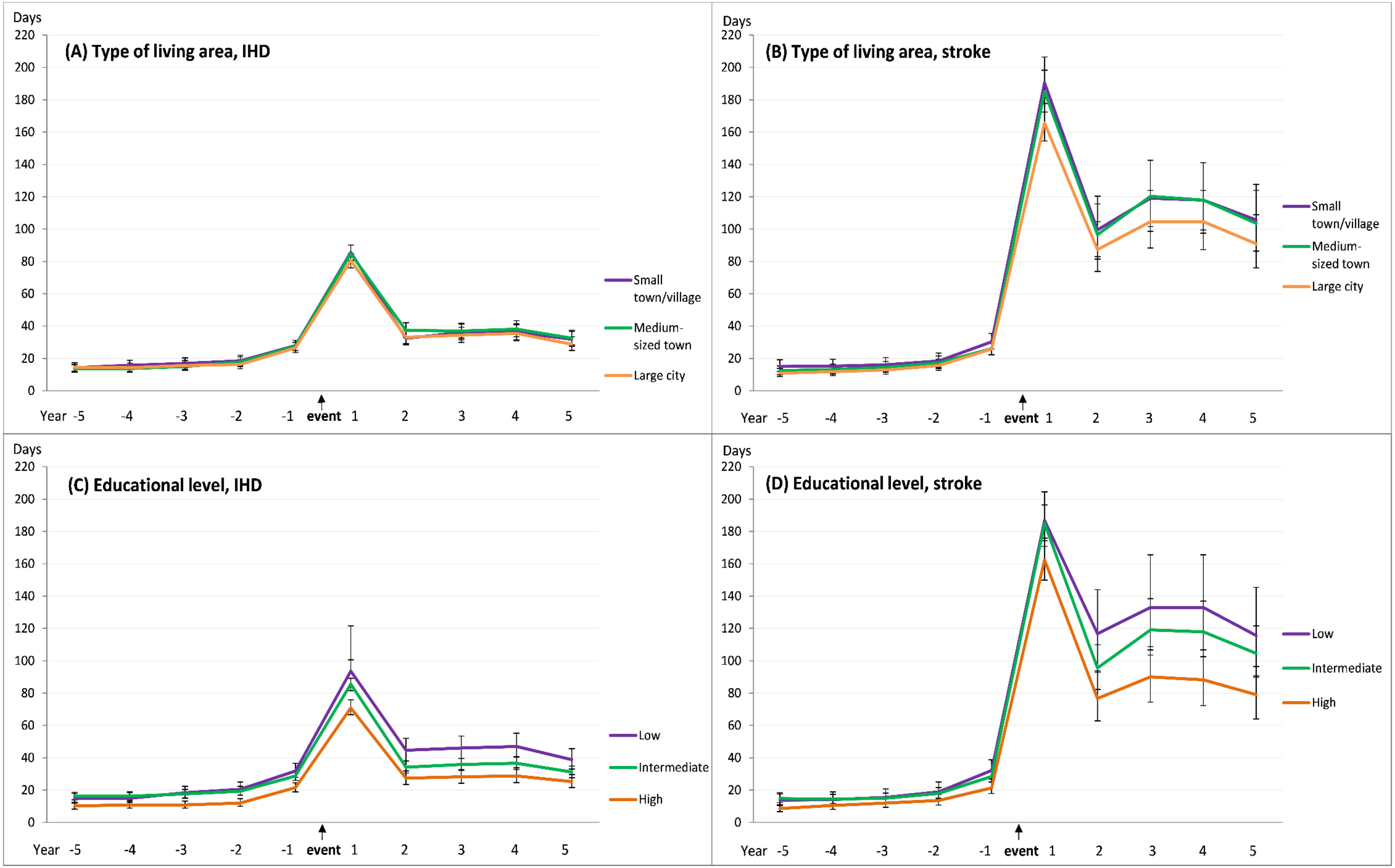

*Type of living area stratified analysis adjusted for age, sex, and education; education-stratified analysis adjusted for age and sex.
